# Pulse Plasma Sintering of NiAl-Al_2_O_3_ Composite Powder Produced by Mechanical Alloying with Contribution of Nanometric Al_2_O_3_ Powder

**DOI:** 10.3390/ma15020407

**Published:** 2022-01-06

**Authors:** Katarzyna Konopka, Justyna Zygmuntowicz, Marek Krasnowski, Konrad Cymerman, Marcin Wachowski, Paulina Piotrkiewicz

**Affiliations:** 1 Faculty of Materials Science and Engineering, Warsaw University of Technology, 141 Woloska St., 02-507 Warsaw, Poland; Katarzyna.Konopka@pw.edu.pl (K.K.); marek.krasnowski@pw.edu.pl (M.K.); konrad.cymerman.dokt@pw.edu.pl (K.C.); paulina.piotrkiewicz.dokt@pw.edu.pl (P.P.); 2 Faculty of Mechanical Engineering, Military University of Technology, 2 Gen. S. Kaliskiego St., 00-908 Warsaw, Poland; marcin.wachowski@wat.edu.pl

**Keywords:** NiAl-Al_2_O_3_ composites, ultrafine-grained Al_2_O_3_, Pulse Plasma Sintering (PPS)

## Abstract

NiAl-Al_2_O_3_ composites, fabricated from the prepared composite powders by mechanical alloying and then consolidated by pulse plasma sintering, were presented. The use of nanometric alumina powder for reinforcement of a synthetized intermetallic matrix was the innovative concept of this work. Moreover, this is the first reported attempt to use the Pulse Plasma Sintering (PPS) method to consolidate composite powder with the contribution of nanometric alumina powder. The composite powders consisting of the intermetallic phase NiAl and Al_2_O_3_ were prepared by mechanical alloying from powder mixtures containing Ni-50at.%Al with the contribution of 10 wt.% or 20 wt.% nanometric aluminum oxide. A nanocrystalline NiAl matrix was formed, with uniformly distributed Al_2_O_3_ inclusions as reinforcement. The PPS method successfully consolidated NiAl-Al_2_O_3_ composite powders with limited grain growth in the NiAl matrix. The appropriate sintering temperature for composite powder was selected based on analysis of the grain growth and hardness of Al_2_O_3_ subjected to PPS consolidation at various temperatures. As a result of these tests, sintering of the NiAl-Al_2_O_3_ powders was carried out at temperatures of 1200 °C, 1300 °C, and 1400 °C. The microstructure and properties of the initial powders, composite powders, and consolidated bulk composite materials were characterized by SEM, EDS, XRD, density, and hardness measurements. The hardness of the ultrafine-grained NiAl-Al_2_O_3_ composites obtained via PPS depends on the Al_2_O_3_ content in the composite, as well as the sintering temperature applied. The highest values of the hardness of the composites were obtained after sintering at the lowest temperature (1200 °C), reaching 7.2 ± 0.29 GPa and 8.4 ± 0.07 GPa for 10 wt.% Al_2_O_3_ and 20 wt.% Al_2_O_3_, respectively, and exceeding the hardness values reported in the literature. From a technological point of view, the possibility to use sintering temperatures as low as 1200 °C is crucial for the production of fully dense, ultrafine-grained composites with high hardness.

## 1. Introduction

Ceramics are combined with metals and intermetallic materials to produce composites. Various metals are used, such as V, Mo, Cu, Ni, Ti, or Fe [1,2,3,4,5,6,7], along with intermetallics such as NiAl [8,9] or Ni_3_Al [10]. Such materials have been developed because of their desirable properties. Improving the fracture toughness of the composites with the ceramic matrix is crucial [11,12]. Furthermore, composites of intermetallic–ceramic systems can offer excellent combinations of properties, e.g., low density and relatively high hardness and strength. The presence of nanocrystalline or ultrafine structure in composites may further enhance their desired properties [13,14]

Powder consolidation methods are the most commonly used methods for making ceramic matrix composites. In these methods, ceramic and metal powders are mixed together, pressed, and sintered. However, composite bulk material can also be achieved by consolidating previously prepared composite powders. One of the methods of synthesis of such materials is mechanical alloying (MA) [15,16], which can be used to prepare composite powders—especially intermetallic matrix composites [17,18]. During high-energy ball milling of selected metal powders, the chemical reaction and phase transformation take place, forming an intermetallic compound. To prepare the intermetallic–ceramic composite powder, the MA process begins with metals and ceramic powders. Consequently, the intermetallic compound is randomly located in the ceramic matrix, or the ceramic particles are uniformly distributed within the intermetallic matrix. Consolidation of such prepared composite powders should also enable the achievement of a uniform distribution of the intermetallic material in the ceramic matrix, or vice versa. Nevertheless, preparation of high-quality, fully dense composites of intermetallic–ceramic systems requires a high sintering temperature. Simultaneously, sintering at elevated temperatures mostly leads to grain growth of the initial powder, which is especially a drawback when the nanometric powders are used for consolidation.

In the present article, NiAl-Al_2_O_3_ composite powders were obtained by the method of MA using nickel, aluminum, and nanometric alumina powders. Nanometric alumina powder was used for the reinforcement of a synthetized intermetallic matrix; thus, enhancement of the hardness was expected.

Pulse plasma sintering (PPS) was chosen as the method of consolidation of the composite powder. During PPS consolidation, electric pulses generated periodically by a discharged capacitor battery heat the powder; simultaneously, the powder is uniaxially pressed during the process. Electric pulses with microseconds of duration have a current intensity of ~100 kA [19,20]. The short duration of the electric pulse relative to the time interval between the individual pulses makes the temperature achieved during the pulse higher than that achieved during the traditional sintering method [19,20]. Due to the short sintering time in PPS, the rapid sinter allows us to obtain the bulk composite without excessive grain growth [20].

Previously, our own experiments with the fabrication of NiAl-Al_2_O_3_ composite powders and their subsequent compaction by PPS produced positive and interesting results [21]. In these studies, alumina powder with micrometric size was used [19]. In the present work, the nanometric alumina powder was used and mixed with nickel and aluminum powders to prepare the composite powders via MA. The powders produced in this way were consolidated via the PPS method. Using nanocrystalline and ultrafine-grained powders and PPS techniques, which ensure a short sintering time without excessive coarsening of the grains, composites with ceramic particles uniformly distributed in a NiAl intermetallic matrix—both ultrafine-grained—can be fabricated. In the case of ultrafine-grained materials, their mechanical properties are superior to those of their coarse-grained counterparts [22]. However, in order to produce ultrafine-grained composites, the selection of the proper sintering temperature for PPS is a key factor. Hence, firstly, PPS processes of the initial nanometric alumina powder were performed at various temperatures. This allowed us to define the influence of temperature on the quality of consolidation and the intensity of grain growth. Based on the obtained results, the consolidation temperatures of the composite powders were selected. The results of PPS consolidation of pure Al_2_O_3_ powder and composite NiAl-Al_2_O_3_ powders, as well as characterization of bulk samples, were described.

## 2. Materials and Methods

### 2.1. Materials

The initial materials implemented in the investigation were commercially available powders. The ceramic powder was applied in the form of α-Al_2_O_3_—alumina, with the trade name TM-DAR, from Taimei Chemicals (TAIMEI CHEMICALS Co., Ltd., Tokyo, Japan). According to the manufacturer’s specifications, the powder particles had a spherical shape, and their size was in the range of 100 ± 20 nm. In the experiments, nickel and aluminum were used as metallic components. Nickel powder (ABCR GmbH & Co.KG, Karlsruhe, Germany) was characterized by 3–7 µm average particle size. The aluminum powder (ABCR GmbH & Co.KG, Karlsruhe, Germany) was characterized by an average particle size of 44 µm. The powders used for grinding were characterized by purity at the level of 99.99%—aluminum oxide, 99.9%—nickel, and 99.7%—aluminum. The data above on the characteristics of the initial powders are based on the manufacturers’ data. Nevertheless, these characteristics required confirmation through experimental investigation.

### 2.2. Preparation of Samples

The first stage of the work was mechanical alloying to obtain a composite powder based on nickel, aluminum, and aluminum oxide. The primary materials for the milling processes were powder blends containing Ni-50at.%Al with the addition of 10 wt.% and 20 wt.% aluminum oxide. The milling processes were performed on a SPEX 8000D high-energy shaker ball mill (SPEX^®^ SamplePrep, Metuchen, NJ, USA) with an 8:1 ball-to-powder weight ratio. The milling processes and powder sampling were carried out under an argon atmosphere.

In the subsequent steps, samples were fabricated using the PPS technique. The PPS method is also identified as a field-assisted sintering technology (FAST) [23]—a process using the activation of an electric field generated by the flow of an external electric current during sintering [23]. During the PPS process, the high-current forcing pulses come from the discharge of the capacitor bank with a capacity of ~200 μF. This forcing causes a rapid increase in the temperature of the sintered powder. This heating method, which is carried out with simultaneous pressing under pressure, allows the ignition temperature of the SHS reaction to be quickly reached, simultaneously, in the entire volume of the synthesized powders [24]. The heating of the powder occurs with Joule’s heat, which is given off during the passage of current through the consolidated powder, and spark discharges (plasma generation) in the spaces between the powder grains. During the PPS process, four operations can be distinguished, including preparing the working atmosphere, setting the press pressure, sintering, and chamber discharge [24,25]. The first step in the PPS method involves the preparation of the working atmosphere, which involves achieving the required vacuum level (low vacuum 1 Pa, high vacuum 10^−3^ Pa). Then, a pressure force starting from 20 MPa and ending at 80 MPa is applied to the punches in which the die is placed. In this way, the pressing pressure during the process is established. Current pulses with specific parameters flow through the powder in the sintering stage. The process parameters were developed based on previous experimental work. At the last stage of the PPS process, the chamber is discharged, i.e., atmospheric pressure is established within it. Table 1 presents the PPS process parameters for the samples produced.

Samples containing pure alumina were sintered at six different temperatures: 1000 °C, 1100 °C, 1200 °C, 1300 °C, 1400 °C, and 1500 °C. These studies aimed to determine the appropriate sintering temperature for samples prepared from ultrafine-grained alumina powder. Subsequently, based on these tests, sintering of the NiAl-Al_2_O_3_ powders was carried out at temperatures of 1200 °C, 1300 °C, and 1400 °C.

### 2.3. Research Techniques

In the research, different techniques were used to determine the initial powders’ characteristics, and to investigate the powders after mechanical alloying, as well as the specimens fabricated by pulse plasma sintering.

A helium pycnometer (AccuPyc 1340 II, Micromeritics, Norcross, GA, USA) was used to measure the actual density of the Al_2_O_3_, NiAl-10%Al_2_O_3_, and NiAl-20%Al_2_O_3_ powders. The density measurements were calculated using the ASTM D3766 standard [26].

According to the European Standard EN623-2 [27], the Archimedes method was applied to determine the selected properties—such as the density, open porosity, and soaking—of the samples obtained via PPS.

To characterize the phase composition, a Rigaku MiniFlex II diffractometer (Rigaku Corporation, Tokyo, Japan) with a CuK radiation wavelength of l = 1.54178 Å was used, set to the following recording parameters: current 15 mA, voltage 30 kV, step D2θ 0.05°, counting time 3 s, angular range 2θ 23–120°. The Williamson–Hall method was employed to estimate the mean crystallite size. The instrumental broadening was subtracted from the experimental breadth to obtain the physical broadening of each diffraction line.

Observations of the morphology of raw Al_2_O_3_, Ni, Al, NiAl-10%Al_2_O_3_, and NiAl-20%Al_2_O_3_ powders, along with the microstructure of the produced bulk specimens, were performed using a JEOL JSM-6610 scanning electron microscope (Tokyo, Japan) equipped with secondary electron (SE) and backscattered electron (BSE) detectors. A voltage of 15 kV was applied throughout the observations. Surface microanalysis was conducted using an X-Max-type energy-dispersive X-ray spectrometer (EDS, Oxford, UK) to determine the elemental concentration in the fabricated composites after MA and after consolidation by PPS.

A quantitative microstructural description was carried out with a MicroMeter v.086b computer image analyzer. The raw Al_2_O_3_ powder and Al_2_O_3_ grains in the bulk samples of pure Al_2_O_3_ obtained at different temperatures were investigated [28,29]. A quantitative description of the microstructure of the samples was carried out based on SEM images of randomly determined areas of the fracture of the samples. This technique provides the ability to obtain knowledge of the actual size of alumina in the specimen. Furthermore, based on stereological analysis, the following shape parameters of the Al_2_O_3_ grains were determined: convexity (W = p/p_c_), a coefficient describing the surface development (R = p/p_c_·d_2_), and elongation (α = d_max_/d_2_), where p is the perimeter of the grain (μm) and p_c_ is the Cauchy perimeter (μm), d_2_ is the diameter of the circle of the same surface as the surface of the analyzed grain (μm), and d_max_ is the maximum diameter of grain projection (μm) [28,29].

The Vickers method was used to determine the hardness of the bulk samples. Hardness was measured on the polished sample surface. The used load was 10 kg, and the holding time was 10 s. In the experiment, an HVS-30T hardness tester (Huatec Group Corporation, Beijing, China) was used. For individual specimens, at least 15 measurements were performed. The indentation sizes were estimated using a Nikon Eclipse LV15ON light microscope (Tokyo, Japan).

## 3. Results and Discussion

### 3.1. Description of the Initial Powders

**Figure 1:**
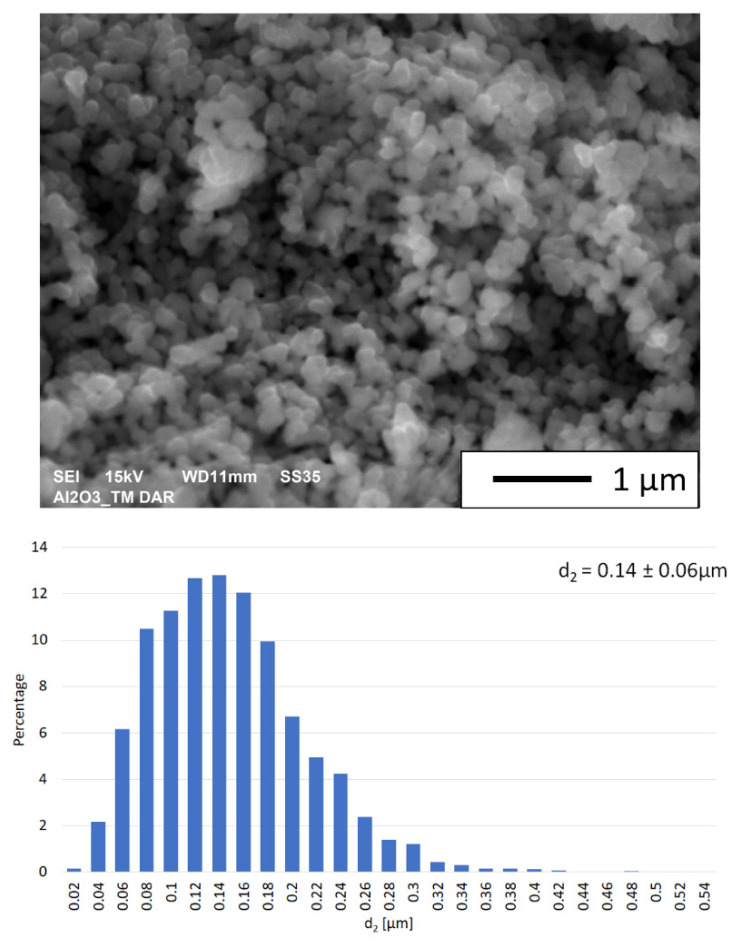
Microstructure and histogram of the particle size distribution of alumina powder.

Figure 1 shows the morphology and a particle size distribution histogram of the alumina powder. Microscopic observations reveal that the Al_2_O_3_ powder used is spherical (Figure 1). The observations showed that alumina tends to form agglomerates. Data from the literature [30] show that nanometric powders, which characterize a high surface-area-to-single-particle volume ratio, can form agglomerates. Analysis of the obtained histogram shows that the alumina particle size distribution is unimodal (Figure 1). The average particle size was determined to be 0.140 µm, with a standard deviation equal to 0.06 µm. The density measured using a helium pycnometer for alumina was 3.9828 g/cm^3^. The density value obtained is close to the density reported by the manufacturer.

**Figure 2:**
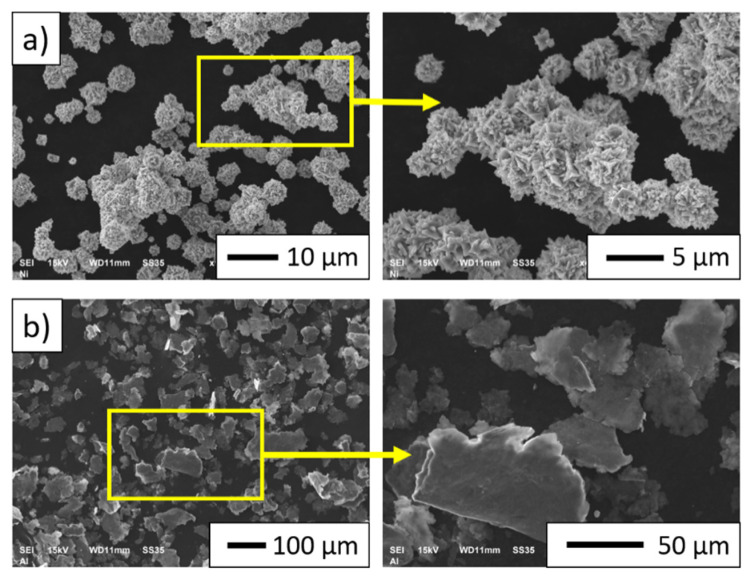
Morphology of metallic powders: (**a**) nickel; (**b**) aluminum.

Figure 2 shows the morphology of the metallic powders used in the investigation. Based on microscopic observations, it was found that the nickel particles are characterized by micrometric size, a spherulite shape with a lot of cavities, and an irregular surface (Figure 2a). Microscopic observations showed that the aluminum particles have a regular flat surface with an elongated shape (Figure 2b).

### 3.2. Description of NiAl-Al_2_O_3_ Composite Powders

**Figure 3:**
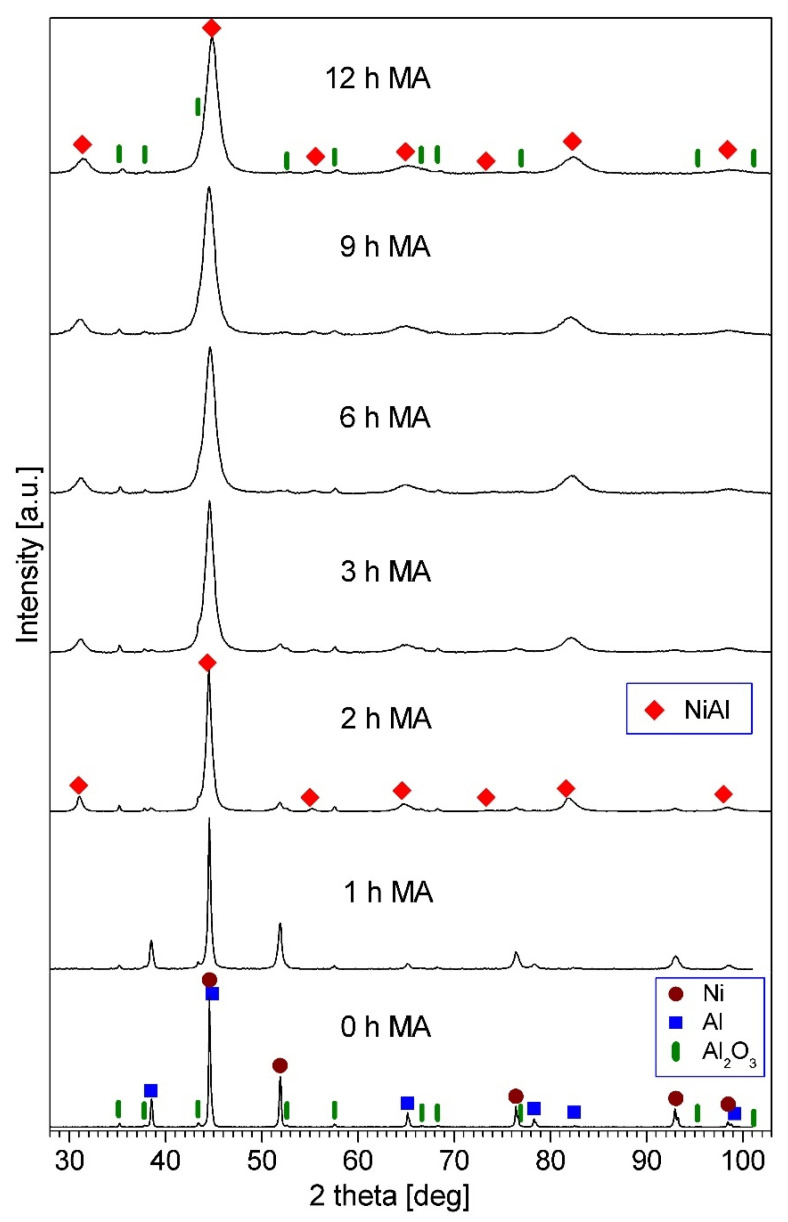
XRD patterns of the (Ni-50at.%Al) + 10wt.%Al_2_O_3_ powder mixture milled for the times quoted.

Figure 3 shows the XRD patterns of the (Ni-50at.%Al) + 10wt.%Al_2_O_3_ sample after various milling times. In the early stage of mechanical alloying, a new phase was developed, corresponding to the appearance of new peaks in the XRD spectra for the two h-milled powders; these peaks correspond to a NiAl intermetallic phase. At the same time, the intensity of the Ni and Al peaks decreased significantly, and these peaks vanished with increasing milling time. The observed phase development (reaction between Ni and Al, and creation of the NiAl phase) is analogous to that previously described for mechanical alloying of a Ni-50at.%Al powder mixture carried out using the same ball mill [31]. The diffraction peaks of alumina remain constantly present in the XRD patterns. The phase composition of the milling product after 12 h is the NiAl intermetallic phase and Al_2_O_3_. For the Ni-50at.%Al-20wt.%Al_2_O_3_ sample, a similar phase evolution was observed; however, it occurred more slowly, and the process required 15 h of milling to be completed. The influence of the amount of reinforcing phase on the rate of the phase evolution and NiAl phase formation during mechanical alloying was reported recently [17]. In the case of ball milling of Ni-Al-B powder mixtures, it was found that with the increase in the amount of boron in the mixture, the creation of the NiAl phase required a longer milling time [17].

As milling time increased, the NiAl diffraction peaks broadened (Figure 3). This widening of the peaks was due to the decrease in the size of the NiAl crystallites, and the increased lattice microstrains in this intermetallic phase [32]. In the final milling product, the mean crystallite size of the NiAl phase was 14 nm and 11 nm for the samples containing 10% and 20% Al_2_O_3_, respectively. The mean crystal size was estimated by the Williamson–Hall method; in this method, it is assumed that the physical broadening of diffraction lines (β) is the sum of broadening resulting from the small size of crystallites (below 0.1 µm) (β_c_) and the presence of lattice microstrains (β_s_) [32]. In the Williamson–Hall method, considering the width of peaks as a function of 2θ allows one to estimate the mean crystallite size and the mean lattice microstrains separately. For the Williamson–Hall analysis, the instrumental broadening was subtracted from the experimental breadth to obtain the physical broadening of each diffraction line.

**Figure 4:**
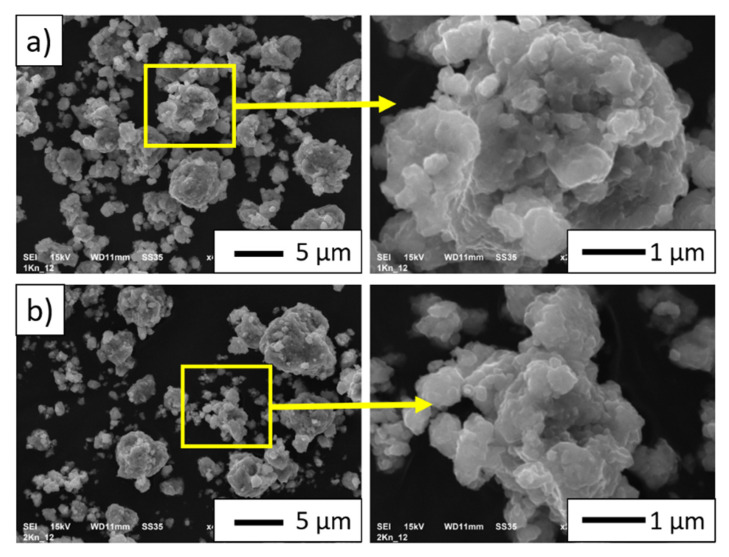
Microphotographs of the powder particles of the mechanic alloying products: (**a**) NiAl-10%Al_2_O_3_ powder; (**b**) NiAl-20%Al_2_O_3_ powder.

Figure 4 shows the SEM images of the composite powder particles after the mechanical alloying: (a) NiAl-10%Al_2_O_3_ powder; (b) NiAl-20%Al_2_O_3_ powder. The observation reveals that the composite powders are characterized by the tendency to create spherical agglomerates with sizes ranging from below 1 µm up to 20 µm. The density measured using a helium pycnometer for NiAl-10%Al_2_O_3_ powder was 5.4571 g/cm^3^, while for the NiAl-20%Al_2_O_3_ powder it was 5.1858 g/cm^3^.

**Figure 5:**
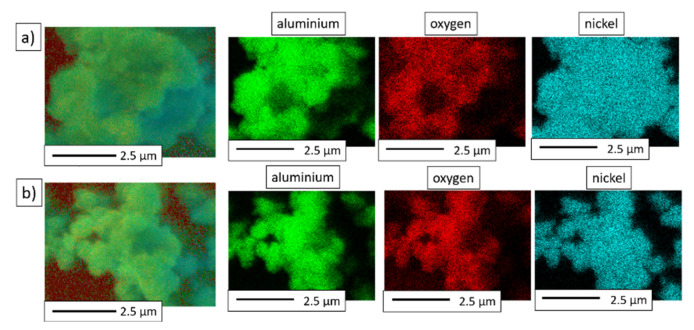
EDS maps of the powder particles of the MA products: (**a**) NiAl-10%Al_2_O_3_ powder; (**b**) NiAl-20%Al_2_O_3_ powder.

The EDS maps of the mechanical alloying products are shown in Figure 5. The presence of three elements—aluminum, oxygen, and nickel—was found. Furthermore, the EDS of the areas studied revealed that NiAl-10%Al_2_O_3_ powder (Figure 5a) contained 15.69 at.% oxygen, 42.96 at.% aluminum, and 41.35 at.% nickel. In the case of NiAl-20%Al_2_O_3_ powder (Figure 5b), it was found that the powder contained 25.70 at.% oxygen, 43.29 at.% aluminum, and 31.01 at.% nickel.

### 3.3. Characterization of Al_2_O_3_ Samples after PPS Consolidation

**Figure 6:**
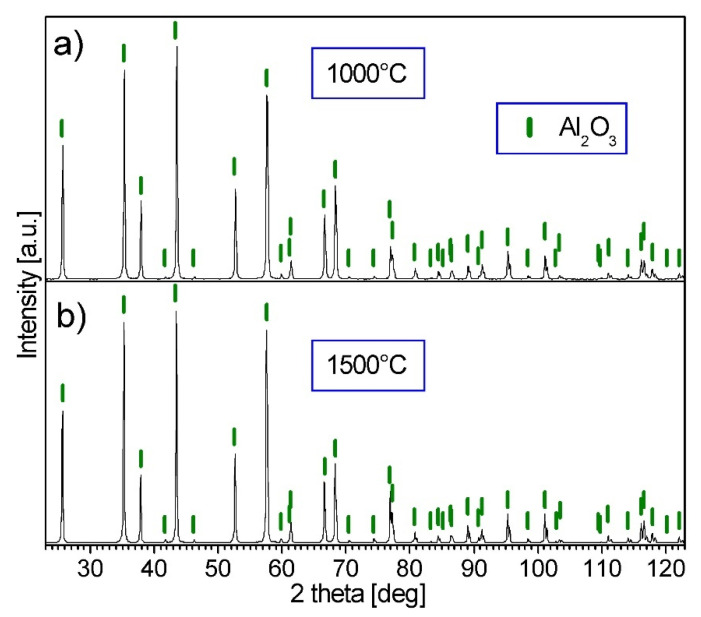
XRD patterns of the A_2_O_3_ powder after consolidation by PPS: (**a**) at 1000 °C; (**b**) at 1500 °C.

The X-ray phase analysis of the alumina powder consolidated at various temperatures confirmed the presence of only the Al_2_O_3_ phase in the material. Figure 6 presents XRD patterns of the samples consolidated at 1000 °C and 1500 °C as examples.

Selected properties of the Al_2_O_3_ samples after PPS consolidation are described in Table 2. The results obtained showed that the samples sintered at a temperature of 1000 °C had a relative density of 74.39 ± 2.45%, while the density of the Al_2_O_3_ samples sintered at 1100 °C was equal to 89.64 ± 1.35%. The sintered samples at temperatures from 1200 °C to 1500 °C were characterized by a relative density close to 100%. Table 2 does not include the open porosity values for sintered samples at temperatures from 1200 °C to 1500 °C, because the open porosity and soaking are negligible for samples with very high densities (>99%). This is consistent with sintering theory, because there should be no open pores at high relative densities; therefore, the absorbency would be zero.

**Figure 7:**
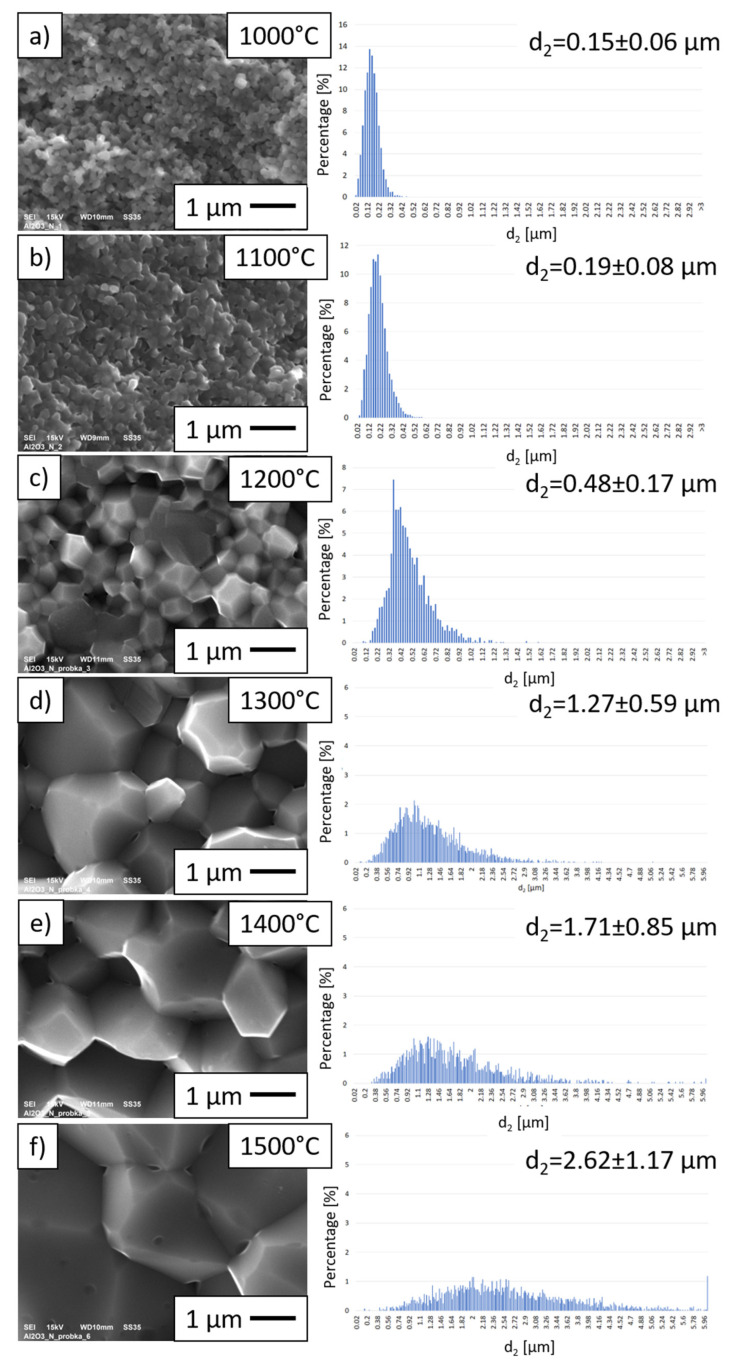
The fracturing of Al_2_O_3_ samples sintered by the PPS method (**a**) at 1000 °C; (**b**) at 1100 °C; (**c**) at 1200 °C; (**d**) at 1300 °C; (**e**) at 1400 °C; (**f**) at 1500 °C, and histograms showing the size distribution of Al_2_O_3_ grains.

Figure 7 shows examples of SEM observation of the fractures of the Al_2_O_3_ samples sintered at different temperatures by the PPS method, together with histograms showing the size distribution of the Al_2_O_3_ grains in the tested samples. Histograms revealed that the alumina samples manufactured by PPS at 1000 °C were characterized by the smallest average grain size. The results showed that the samples sintered at 1000 °C had an average Al_2_O_3_ grain size of 0.15 ± 0.06 µm. For these specimens, Al_2_O_3_ grains were in the size range of 0.02 to 0.46 µm. It was found that sintering at 1000 °C does not cause grain growth of the original powder but, on the other hand, does not allow good-quality compacting of the samples. For the samples consolidated at 1100 °C, a non-significant increase in the size of Al_2_O_3_ grains was observed. For these samples, an average grain size of Al_2_O_3_ of 0.19 ± 0.08 µm was noted. In the case of the samples sintered at temperatures of 1000 °C and 1100 °C, weak consolidation of the samples was observed.

For the samples manufactured by PPS at 1200 °C, the average Al_2_O_3_ size was 0.48 ± 0.17 µm. For the samples sintered at 1200 °C, an over-threefold increase in the size of the Al_2_O_3_ grains was observed compared to the original powder size (0.14 ± 0.06 µm). For the samples obtained at 1300 °C, the average grain size was 1.27 ± 0.59 µm. Sintering at 1300 °C caused the Al_2_O_3_ grains to grow ninefold compared to raw powder, while sintering at a temperature of 1400 °C resulted in a 12-fold increase in Al_2_O_3_ grains compared to with raw alumina. For the samples obtained at 1400 °C, the grain size ranged from 0.02 µm to 5.99 µm, and the average size was 1.71 ± 0.85 µm. The highest growth of Al_2_O_3_ grains was observed with the starting powder for the samples sintered at a temperature of 1500 °C. Histograms showed that the average grain size of Al_2_O_3_ was in the range of 0.42 µm to 5.98 µm. The average size of the alumina grains for the samples at 1500 °C was 2.62 ± 1.17 µm—more than 18 times the size of Al_2_O_3_ grains in the starting powder.

SEM observations of the fractures of the alumina samples sintered at temperatures of 1200 °C (Figure 7c), 1300 °C (Figure 7d), 1400 °C (Figure 7e), and 1500 °C (Figure 7f) showed a significant effect of the temperature on the grain growth of Al_2_O_3_ in the samples during the PPS process; this was confirmed by analysis of histograms. In all of these samples, complete sintering of the alumina grains was observed; moreover, this was confirmed by the high density results (Table 2).

The parameters characterizing the shape factors of alumina grains in all samples are shown in Table 3. Based on the obtained results, it can be concluded that a similar shape characterizes the Al_2_O_3_ grains sintered at 1000 °C and 1100 °C. This is evidenced by the similar values of parameters, i.e., convexity, the curvature of grain boundaries, and elongation. Moreover, the average Al_2_O_3_ grain size values are close to the starting size of the alumina powder. Analysis of parameters such as convexity, the curvature of grain boundaries, and elongation showed that grains in all samples were similar.

A high sintering temperature is essential to obtaining good-quality samples; on the other hand, the use of a high sintering temperature causes grain growth. Accordingly, the applied temperature should be high enough to allow good sintering, but at the same time, it must not be so high that the grains do not grow excessively. First, experiments for pure Al_2_O_3_ were carried out in the research, enabling the selection of the appropriate temperature for NiAl-Al_2_O_3_ sintering. For further research, the temperature of 1500 °C was not selected, despite the density of samples being close to 100% due to the excessive growth of Al_2_O_3_ grains.

**Figure 8:**
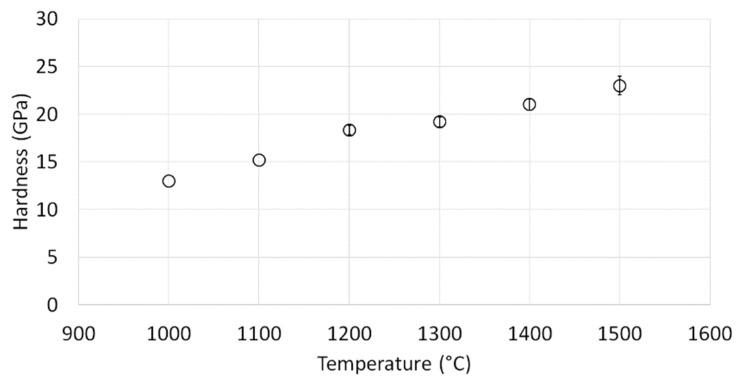
Hardness values for Al_2_O_3_ samples.

Figure 8 shows the obtained hardness values for the alumina samples. The values obtained varied from 13 GPa to 23 GPa, and were correlated with the sintering temperatures applied during their preparation. Consequently, the samples sintered at 1000 °C were characterized by the lowest hardness values, with a hardness equal to 13.00 ± 0.38 GPa, while the samples sintered at the highest temperature of 1500 °C were characterized by the highest hardness values, amounting to 23.00 ± 0.97 GPa.

The observed correlation between the hardness and the sintering temperature of Al_2_O_3_ ceramics confirmed the increased compaction quality of samples sintered at higher temperatures. This effect is not clearly visible in the density values of materials, which are similarly high for sintering at 1300, 1400, and 1500 °C; however, it is visible during SEM observations of the fracture surface of samples. For the sample sintered at 1500 °C—in which the largest grain size was observed and, at the same time, the highest hardness was achieved—good compaction of grains prevented its pulling out (Figure 7f). It should be noted that the grains of Al_2_O_3_ grow to more than 18 times the size of the initial Al_2_O_3_ grains. However, it can be concluded that the increase in temperature, together with the better developed interfaces between grains, results in high hardness.

The obtained hardness values exceeded those for the similarly formed Al_2_O_3_ ceramics from the micrometric powders studied in our previous work [21]. While the maximal hardness attained previously for Al_2_O_3_ sintered at the highest temperature of 1500 °C amounted to 15.30 ± 0.87 GPa [21], the corresponding sample of ultrafine-grained Al_2_O_3_ ceramics in the present study (sample sintered at 1500 °C) exceeded this value by 67%

Moreover, the achieved results correlate well with the results presented in the literature regarding dense Al_2_O_3_ ceramics. In the work carried out by Yuan et al. [33], oscillatory pressure sintering (OPS) and hot pressing at various temperatures in the range from 1100 °C to 1500 °C were used to produce high-density Al_2_O_3_ ceramics. The demonstrated hardness–temperature correlation was analogous to that observed in our study, as the hardness increased with increasing temperature, which was associated with higher compaction quality. Regarding the higher sintering temperatures, the hardness of samples obtained via the OPS method, with a maximum value at 1500 °C of approximately 24 GPa, stands in good correlation with the results achieved in our study. In the case of the lower sintering temperatures, the materials analyzed were characterized by lower hardness, with values below 10 GPa for 1100 °C. In comparison, the hardness of the samples made by the hot-pressing method was generally lower throughout the entire range [33].

Furthermore, in the study by Sun et al., where a combination of uniaxial pressing and pressureless sintering was employed to produce Al_2_O_3_ ceramics from nanospheres, the hardness of the ceramics sintered at 1550 °C amounted to 23.00 ± 1.4 GPa—a value very similar to our results. The relationship between hardness and sintering temperature was similarly observed, attributed to the observed correlation between the densification of the material and the size of the grains [34].

Our results showed that hardness of the specimens produced from the pure nanometric Al_2_O_3_ powder, as expected, was significantly higher than that of the NiAl-Al_2_O_3_ composites (results presented in Section 3.3).

### 3.4. Characterization of NiAl-Al_2_O_3_ Samples after PPS Consolidation

**Figure 9:**
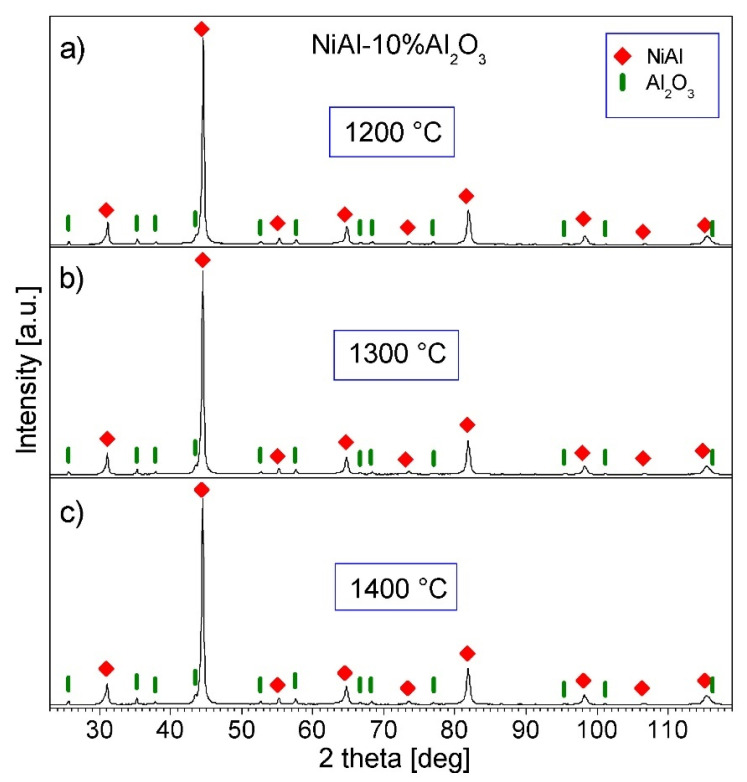
XRD patterns of the NiAl-10%Al_2_O_3_ samples consolidated by PPS: (**a**) at 1200 °C; (**b**) at 1300 °C; (**c**) at 1400 °C.

**Figure 10:**
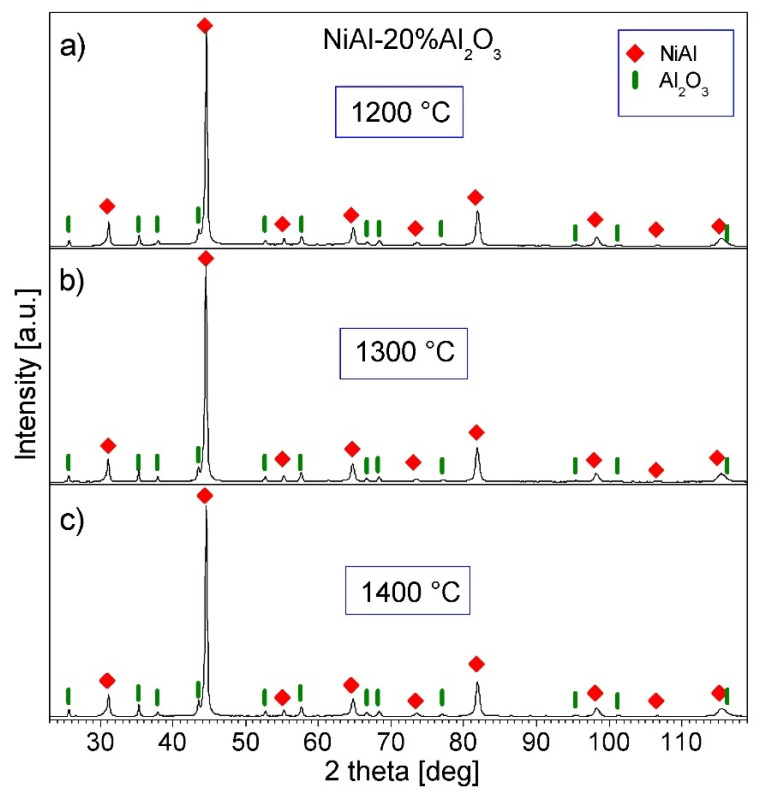
XRD patterns of the NiAl-20%Al_2_O_3_ samples consolidated by PPS: (**a**) at 1200 °C; (**b**) at 1300 °C; (**c**) at 1400 °C.

Figure 9 and Figure 10 show the XRD patterns of the NiAl-Al_2_O_3_ composite powders consolidated at various temperatures. Only NiAl and Al_2_O_3_ diffraction peaks are present in the patterns, showing that no new phases are formed during the PPS process. The width of the NiAl diffraction patterns is smaller than the breadth of the NiAl peaks in the powders before the consolidation. The revealed sharpened peaks indicate grain growth in the NiAl phase during the PPS process. The mean crystallite size of the NiAl phase estimated by the Williamson–Hall method is in the range of 98–130 nm, and increases with the increase in the consolidation temperature. Thus, the estimated grain size is just above 100 nm, so it is at the limit of the applicability of the Williamson–Hall method, and may be affected by error. It can be concluded that the NiAl intermetallic phase in the consolidated samples has an ultrafine grain size.

**Figure 11:**
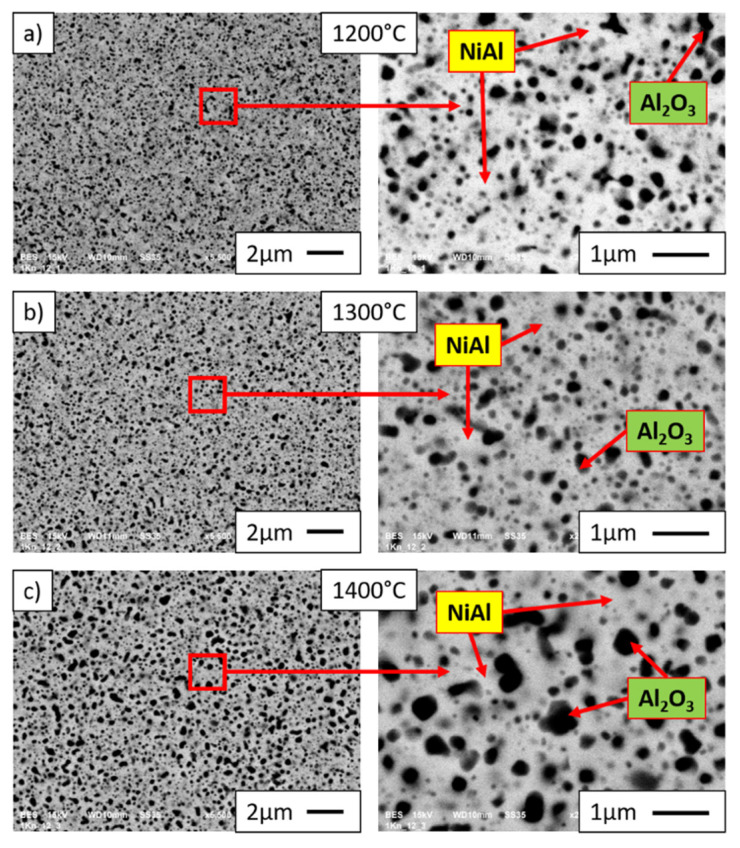
SEM images of microstructures of NiAl-10%Al_2_O_3_ samples after sintering by PPS at (**a**) 1200 °C, (**b**) 1300 °C, and (**c**) 1400 °C.

**Figure 12:**
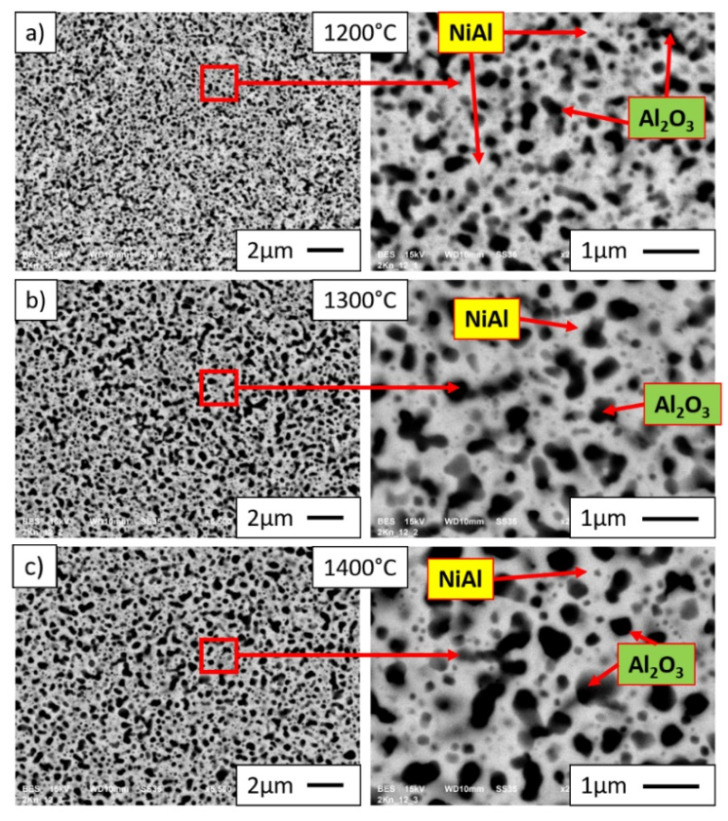
SEM images of microstructures of NiAl-20%Al_2_O_3_ samples after sintering by PPS at (**a**) 1200 °C, (**b**) 1300 °C, and (**c**) 1400 °C.

The SEM images obtained in the BSE (backscattered electron) mode presented in Figure 11 and Figure 12 show the microstructure of sintered NiAl-Al_2_O_3_ composites with different alumina phase contents (10%—Figure 11; 20%—Figure 12) and different temperatures of the sintering process; red-marked areas correspond to magnified areas. The Al_2_O_3_ phase is revealed as dark grey areas in BSE mode, while the NiAl matrix is shown as light gray areas. Observation revealed Al_2_O_3_ inclusions in the NiAl matrix, which were fairly uniformly distributed throughout the volume of the composite samples. A similar structure was obtained by consolidation of milled Ni-Al-B powders; in this case, composites with boron particles uniformly distributed in the nanocrystalline NiAl matrix were produced [17]. The analysis of the SEM results did not reveal areas characterized by enrichment or lack of the intermetallic phase, regardless of the content of the alumina phase and the temperature of sintering. The composite with the highest average Al_2_O_3_ inclusions was obtained during sintering at a temperature of 1400 °C for the sample with 20% alumina content. Results of investigation revealed a lack of cracks and pores in the microstructure of the samples, independent of Al_2_O_3_ phase content, confirming good quality of the consolidation process performed. Moreover, the density of samples reached 100%.

**Figure 13:**
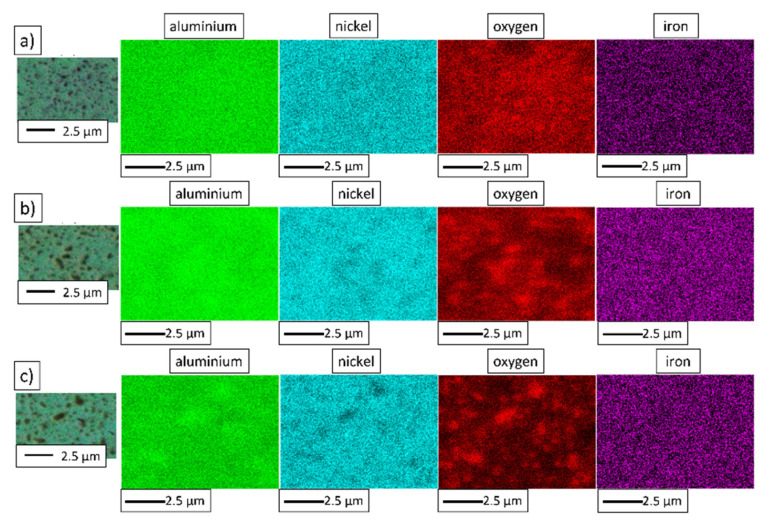
The NiAl-10%Al_2_O_3_ samples’ elemental distribution maps after sintering by PPS at different temperatures: (**a**) 1200 °C; (**b**) 1300 °C; (**c**) 1400 °C.

**Figure 14:**
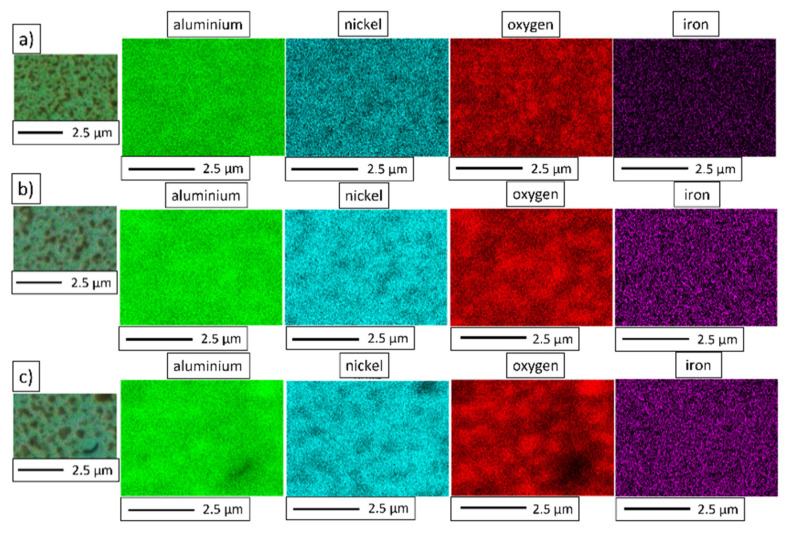
The NiAl-20%Al_2_O_3_ samples’ elemental distribution maps after sintering by PPS at different temperatures: (**a**) 1200 °C; (**b**) 1300 °C; (**c**) 1400 °C.

To reveal the chemical composition of the composite samples, energy-dispersive X-ray spectroscopy (JEOL Ltd., Tokyo, Japan) was applied. Maps of the elemental distribution of the NiAl-10%Al_2_O_3_ and NiAl-20%Al_2_O_3_ composites were produced—the maps obtained from the surface of the NiAl-10%Al_2_O_3_ sample are shown in Figure 13, while the maps for NiAl-20%Al_2_O_3_ are presented in Figure 14. The results of the EDS analysis confirmed the chemical composition of the light and dark areas observed in the SEM images as a NiAl matrix and Al_2_O_3_, respectively. Contamination of the composites by iron was shown by the EDS results for the composites, regardless of the content of the alumina phase. The presence of iron is the result of contamination of the powders from steel milling tools, which is typical for mechanical alloying processes [16].

The hardness measurements for NiAl-Al_2_O_3_ composites indicated that the hardness strongly depends on the Al_2_O_3_ content in the composite, as well as on the sintering temperature applied.

**Figure 15:**
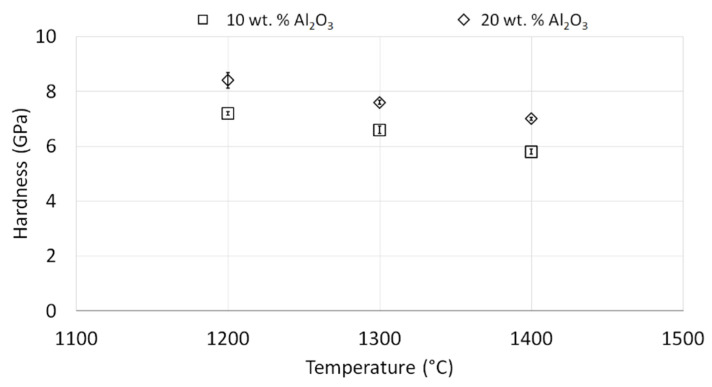
Hardness values for NiAl-10%Al_2_O_3_ samples and NiAl-20%Al_2_O_3_ samples.

Figure 15 shows hardness values for thr NiAl-10%Al_2_O_3_ and NiAl-20%Al_2_O_3_ samples. The hardness of the composites containing 20 wt.% Al_2_O_3_ was in the range of 7.0–8.4 GPa, while for the composites with 10 wt.% Al_2_O_3_ the hardness values varied from 5.8 to 7.2 GPa. Hence, it can be concluded that the addition of Al_2_O_3_ to the composites enhances their hardness, which was predicted as the consequence of hard reinforcement.

However, regardless of Al_2_O_3_ content in the structure, the specimens sintered at the lowest temperature (1200 °C) were characterized by the highest hardness in both series, reaching 7.2 ± 0.29 GPa and 8.4 ± 0.07 GPa for 10 wt.% Al_2_O_3_ and 20 wt.% Al_2_O_3_, respectively. The hardness variation by the applied sintering temperature was lower for the samples with 20 wt.% Al_2_O_3_ content than for the samples with a lower amount of Al_2_O_3_.

Moreover, the hardness of NiAl-Al_2_O_3_ composites decreased with increasing sintering temperature, while the opposite was the case for sintered Al_2_O_3_ samples. This phenomenon could be explained by various structures of the compared samples, the developed interfaces, and/or the growth of NiAl and Al_2_O_3_ grains.

The intermetallic matrix in all samples (1200 °C, 1300 °C, 1400 °C) has an ultrafine grain size (98 to 130 nm); however, the growth of NiAl grains is correlated with the temperature of sintering. At the same time, the growth of Al_2_O_3_ grains is more intensive, as was expected after grain growth investigation of pure Al_2_O_3_ powder consolidated by PPS. SEM observations revealed the growth of Al_2_O_3_ inclusions with the increase in the temperature of sintering (Figure 11 and Figure 12). These changes of both matrix and reinforcement grains contribute to the hardness. However, the new interfaces between NiAl and Al_2_O_3_ grains appeared in composites. Moreover, Al_2_O_3_ inclusions are separated by NiAl matrix grains (Figure 13 and Figure 14); thus, we can predict the effect of hardening by Al_2_O_3_ inclusions. With smaller inclusions, higher hardening should be expected. The contribution of the microstructure of composites to crack propagation should be also considered; investigations in this area are in progress.

The hardness values obtained for lower Al_2_O_3_ content (10 wt.%) were practically identical to those presented in the previous research conducted by the authors of this work [21]. The composite with 20 wt.% Al_2_O_3_ after sintering at 1400 °C reached the highest value compared to the corresponding sample from the previous work [19]. The hardness values of the NiAl-Al_2_O_3_ composites with 10 wt.% and 20 wt.% Al_2_O_3_ content sintered at 1400 °C were the same, at 5.8 GPa [21]; however, in the previous study, micrometric alumina was used as the initial powder [21].

Regarding the material analyzed by Albiter et al. [35], the hardness of NiAl produced by a combination of mechanical alloying with vacuum hot pressing at 1200 °C and 1500 °C was equal to 5.1 GPa and 5.0 GPa, respectively. Similar results were obtained by Biranvand et al. [36], where the hardness of NiAl fabricated by combining mechanical alloying and spark plasma sintering at 1000 °C reached 5.63 ± 0.07 GPa. The hardness of nanocrystalline NiAl produced by mechanical alloying followed by high-pressure hot-pressing consolidation was 9.53 GPa [31]. According to Kaliński et al. [37], the hardness of NiAl samples obtained by a combination of mechanical alloying and hot pressing amounted merely to 3.08 ± 0.10 GPa for those sintered at 1400 °C; in the same study, however, it was confirmed that the addition of Al_2_O_3_ to the structure could improve the mechanical characteristics of the resulting composite, including hardness. The hardness of the analogically prepared NiAl with the addition of 20 vol.% Al_2_O_3_ was found to be equal to 4.29 ± 0.5 GPa [37] A similar relationship was observed in research carried out by Zhu et al. [38], where the addition of Al_2_O_3_ caused an increase in the microhardness of the material up to a value reached for ~10 wt.% Al_2_O_3_ in the composite. The decrease in hardness with further increasing Al_2_O_3_ content was associated with an increase in the material’s porosity and the appearance of a brittle Ni_2_Al phase in the structure [38]. The favorable effect of Al_2_O_3_ on the hardness of the NiAl matrix composite was also demonstrated in the study conducted by Udhayabanu et al. [39], where the composite with 10 vol.% Al_2_O_3_ was obtained by a combination of reactive milling and subsequent SPS. The composite hardness value of 7.6 GPa was found to result from the presence of nanometric particles of Al_2_O_3_ [9]. The hardness of the composites with a similar structure, but containing boron instead of alumina distributed in the NiAl matrix, ranged from 10.3 GPa to 12.6 GPa, and increased with increasing boron content [17].

Analyzing the available scientific literature, it should be noted that the hardness of NiAl observed in various studies exhibits considerable discrepancies depending on the starting materials applied, the manufacturing method, and/or the sintering temperature.

Bulk NiAl-Al_2_O_3_ composites obtained in the presented research exceeded the hardness values reported in the literature and in the authors’ previous paper [21]. The following factors can be held responsible for these results: the contribution of nanometric initial Al_2_O_3_ powders in the MA process and, consequently, the presence of the ultrafine-grained structures in the composites after sintering, along with the high level of compaction of the obtained materials.

## 4. Conclusions

To the best of our knowledge, this paper represents the first time NiAl-Al_2_O_3_ composite powders have been consolidated by the pulse plasma sintering method after mechanical alloying.

Final NiAl-Al_2_O_3_ composites were obtained from composite powders that were produced by mechanical alloying of powder blends containing Ni-50at.%Al, with the contribution of 10 wt.% and 20 wt.% nanometric aluminum oxide powder. Nanometric initial Al_2_O_3_ powder was used for the reinforcement of synthetized intermetallic matrix powder in MA processes; consequently, the presence of ultrafine-grained structures of composites after PPS sintering enhanced the hardness.

Grain growth and hardness of bulk Al_2_O_3_ after PPS consolidation at various temperatures were analyzed; these studies aimed to determine the appropriate sintering temperature for the composite powders. Subsequently, based on these tests, sintering of the NiAl-Al_2_O_3_ powders was carried out at temperatures of 1200 °C, 1300 °C, and 1400 °C.

Characterization of the morphology and size of initial powders, composite powders and, finally, the grains in samples after PPS consolidations, were carried out in order to describe the correlation between the sintering temperature, structure, and hardness of composites. The hardness of the NiAl-Al_2_O_3_ composites depends on the Al_2_O_3_ content in the material and the sintering temperature applied. The samples sintered at the lowest temperature (1200 °C) were characterized by the highest hardness, reaching 7.2 ± 0.29 GPa and 8.4 ± 0.07 GPa for the composites containing 10 wt.% Al_2_O_3_ and 20 wt.% Al_2_O_3_, respectively. The hardness of the ultrafine-grained NiAl intermetallic matrix composites with Al_2_O_3_ inclusions exceeded the hardness values reported in the literature. The contribution of the intial nanometric Al_2_O_3_ powders in the MA process, and the consequent presence of the ultrafine-grained structures in the composites after sintering and the high level of compaction of the obtained materials, as well as interfaces between NiAl and Al_2_O_3_, are responsible for the high hardness of the bulk composites. The increase in the sintering temperature does not improve the compaction of the composite powders; moreover, it causes the grain growth of Al_2_O_3_ inclusions to activate.

From a technological point of view, the possibility of using a sintering temperature as low as 1200 °C is crucial in order to produce fully dense, ultrafine-grained composites with high hardness.

Investigations of correlations between the microstructures of NiAl-Al_2_O_3_ composites and mechanical properties, crack propagation, and fracture toughness are in progress.

## Figures and Tables

**Table 1:** PPS process parameters for the samples produced.

PPS Process Parameter	Voltage (kV)	Stored Energy (kJ)	Electro-Pulse Repetition (s)	Sintering Temperature (°C)	Heating Rate (°C/min)	Sintering Time (s)	Load (MPa)
Al_2_O_3_ samples	5.2–5.8	4.06–5.05	1–1.3	1000, 1100, 1200, 1300, 1400, 1500	250	180	20–80
NiAl-Al_2_O_3_ composite	4.3	2.77	1.3	1200, 1300, 1400			

**Table 2:** Selected properties of Al_2_O_3_ samples obtained by the PPS technique.

Sintering Temperatures of Al_2_O_3_ Samples	1000 °C	1100 °C	1200 °C	1300 °C	1400 °C	1500 °C
Apparent density (g/cm^3^)	2.95 ± 0.78	3.56 ± 0.43	3.97 ± 0.02	3.98 ± 0.01	3.98 ± 0.01	3.98 ± 0.01
Relative density (%)	74.39 ± 2.45	89.64 ± 1.35	99.92 ± 0.01	99.98 ± 0.01	99.98 ± 0.01	99.98 ± 0.01
Open porosity (%)	32.23 ± 1.03	10.67 ± 0.86	-	-	-	-
Soaking (%)	11.06 ± 0.89	3.62 ± 0.45	-	-	-	-

**Table 3:** The parameters characterizing the shape factors of alumina grains in all samples.

Sintering Temperatures of Al_2_O_3_ Samples	Convexity	Curvature of Grain Boundaries	Elongation
1000 °C	1.08 ± 0.01	1.26 ± 0.03	1.38 ± 0.02
1100 °C	1.09 ± 0.01	1.27 ± 0.01	1.38 ± 0.02
1200 °C	1.06 ± 0.01	1.19 ± 0.01	1.30 ± 0.01
1300 °C	1.06 ± 0.01	1.19 ± 0.01	1.30 ± 0.01
1400 °C	1.07 ± 0.01	1.22 ± 0.02	1.22 ± 0.02
1500 °C	1.06 ± 0.01	1.20 ± 0.02	1.30 ± 0.01

## Data Availability

Data sharing not applicable.
